# Deep Learning Can be Used to Classify the Disease Status of the Canine Middle Ear From Computed Tomographic Images

**DOI:** 10.1111/vru.70065

**Published:** 2025-07-26

**Authors:** Zhixuan Zhao, Oisin Mac Aodha, Carola Riccarda Daniel, Nicolas Israeliantz, Anna Orekhova, Tobias Schwarz, Richard Mellanby, Christopher J. Banks

**Affiliations:** ^1^ School of Informatics The University of Edinburgh Edinburgh UK; ^2^ Royal (Dick) School of Veterinary Studies The University of Edinburgh Edinburgh UK; ^3^ Roslin Institute The University of Edinburgh Edinburgh UK

**Keywords:** automated diagnosis, transfer learning, CT, otitis media

## Abstract

Middle ear disease occurs frequently in dogs. CT has proven to be an excellent diagnostic tool for detecting middle ear structures, helping to achieve rapid and accurate diagnoses. Deep learning techniques are now widely used in CT scan‐based human medical image analysis, providing decision support and diagnostics. However, such techniques are currently underutilized in veterinary radiology. The focus of this study was to develop a deep learning model capable of diagnosing middle ear disease in dogs using CT images. To achieve this with a relatively small dataset, transfer learning and data augmentation techniques were applied. During the experimental phase of the study, we tested 10 binary classification models based on the ResNet architecture, combined with data augmentation and transfer learning, on a dataset consisting of a total of 535 canine CT images. We achieved a classification accuracy of up to 84.7%. The developed classifier, trained on relatively few CT images, can detect normal middle ears and middle ear disease in dogs with over 80% accuracy.

## 1 Introduction

**FIGURE 1:**
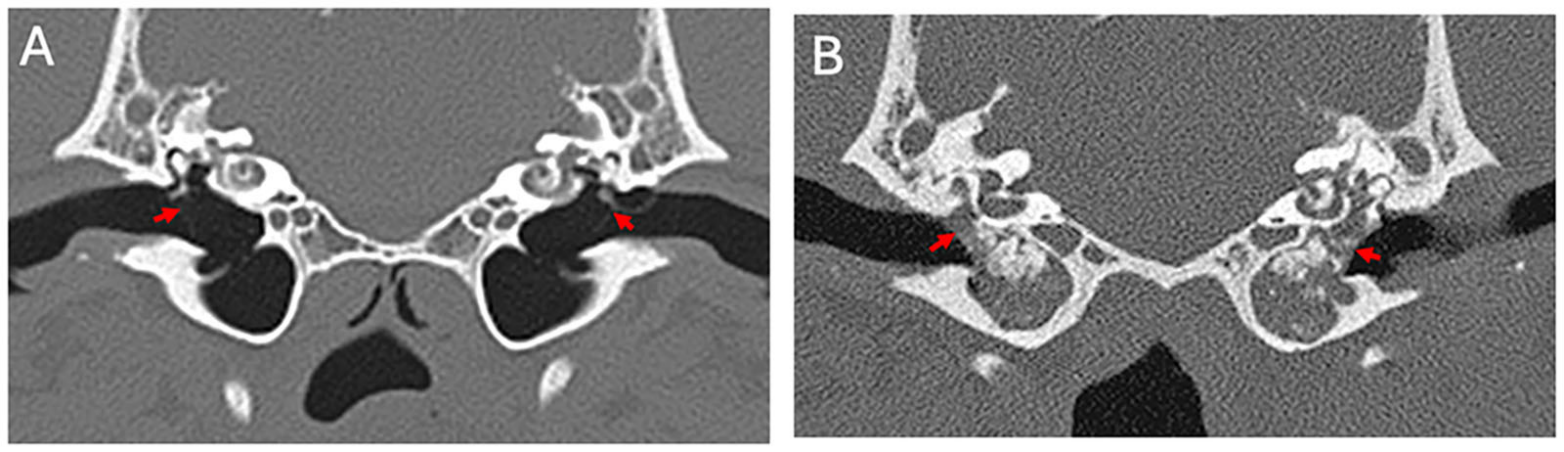
CT images of canine middle ears. (A) Bilaterally normal air‐filled middle ears, thin bulla walls, and partially visible tympanic membranes (arrows). (B) Bilaterally diseased middle ears in which the lumen is filled with soft tissue and mineralized material that is flush with the tympanic membrane (arrows) and moderately thickened bulla walls.

Middle ear disease is a common condition in dogs. It can lead to permanent damage to the tympanic bulla and the neighboring inner ear, pain, and hearing loss, and can also extend into the skull bones and cranial cavity, leading to neurological symptoms. Therefore, misdiagnosis or delayed diagnosis can pose a serious threat to an animal's health and quality of life. The most significant change observed in CT examinations of patients with middle ear disease is the filling of the bulla with fluid or soft tissue material, as illustrated in Figure 1. As a result, CT examination is now widely accepted as an important tool to aid in the diagnosis of canine middle ear disease.

In recent years, the demand for radiology services has grown significantly and has led to excessive demands on the workload for radiologists [1]. This pressure can lead to tiredness and an increased error rate. However, timely reporting of important results is essential for enhancing patient prognosis [2]. The recent rapid growth of artificial intelligence (AI) technology offers the potential for automatic analysis of image data, which may help to reduce radiologists’ workload [3].

Deep learning is one such established AI technology that has already demonstrated impressive performance across a range of image classification tasks, including applications in disease diagnosis [3, 4, 5, 6]. However, most of the medical applications of this work have been focused on human, as opposed to animal, medicine. Appleby and Basran [7] provide a general overview of the state of the field of AI in veterinary medicine. One of the main factors aiding the growth of human medical applications is the abundance of well‐curated and annotated datasets from human imaging. Whilst veterinary images are often well‐curated, digitized, and archived, few are well‐annotated. To address this, for this study, we commissioned the annotation of a set of images that were not previously annotated.

Based on our review of the literature, there have been no attempts to apply deep learning algorithms and techniques for classifying middle ear disease in animals from CT scans. A recent review [8] found that fewer than 40 peer‐reviewed publications used deep learning techniques for veterinary clinical imaging applications, and none of these dealt with cranial CT scans or middle ear diseases. Of note is Wang et al. [6] who diagnosed otitis media in humans.

Convolutional neural networks (CNNs) have demonstrated strong performance in many image recognition challenges [9] and have resulted in impressive performance across a number of difficult image classification problems [10]. In this work, we applied a CNN architecture for the automatic processing of CT scan slices. The CNN architecture used was a residual neural network (ResNet) [10], which has been successfully applied to many visual classification tasks, including multiple applications in medical image analysis [11, 12].

This study aimed to develop an end‐to‐end deep learning model for the automatic diagnosis of middle ear disease, which can be used to assist radiologists in veterinary practice. We approached this study with two main hypotheses:

1. A CNN can be trained to classify middle ears as normal or affected by middle ear disease for use in assisting radiologists in diagnosis in a veterinary setting.
2. A limited training set can be leveraged to train the CNN via the use of data augmentation and transfer learning, and class imbalance can be accounted for by weighting and over‐sampling.

## 2 Materials and Methods

### 2.1 Selection and Description of Subjects

In this analytical study, we developed an automated binary classifier to detect middle ear disease by identifying the presence (i.e., diseased) or absence (i.e., normal) of fluid/soft tissue content in the middle ear cavity on CT scans of subjects.

**FIGURE 2:**
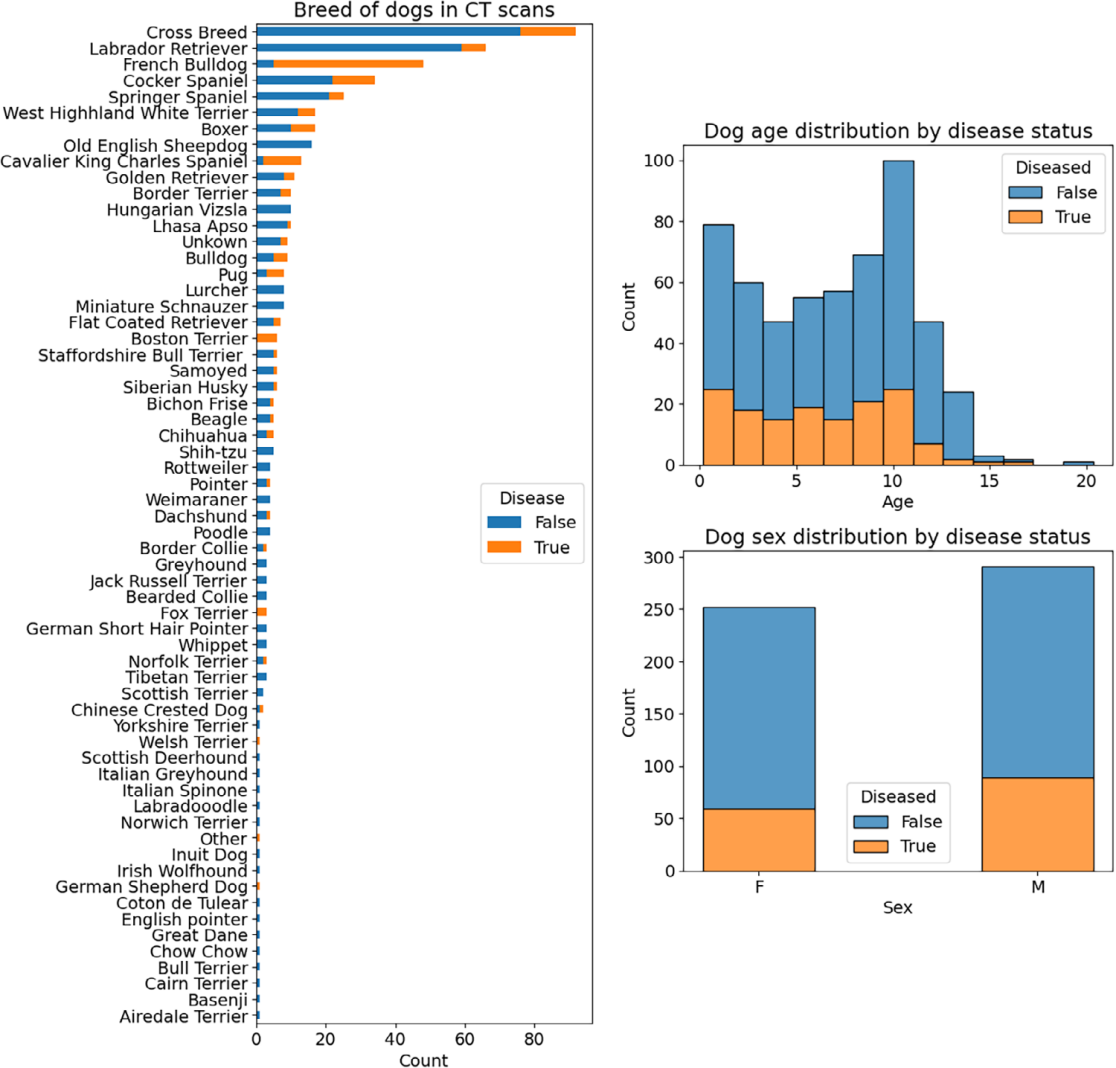
Distribution of dog breeds, ages, and sexes used in the study. Each distribution is divided by disease status as assessed by the examiners.

We used a dataset collected from 535 canine patients at the Royal (Dick) School of Veterinary Studies at The University of Edinburgh, collected between 2009 and 2020. The study protocol and data usage were approved by the institutional veterinary ethical review committee (reference VERC 45.22). All CT scans were acquired using one of two multi‐slice CT scanners (4‐slice‐CT Siemens Volume Zoom 2009—September 2016, 64‐slice‐CT Siemens Definition AS October 2016—2020). CT image acquisition was performed by trained radiographers or radiologists. CT examinations were acquired in ventral recumbency, with a slice width of 1 to 2 mm, 120 kVp, helical mode, using a bone reconstruction kernel (proprietary name *H70s*) from an image acquisition without administration of contrast medium. In total, there were 535 CT head examinations of different breeds and ages of dogs. The distribution across the different breeds is shown in Figure 2. The review of the CT studies was performed by four diagnostic imaging residents and one board‐certified radiologist by consensus. They are termed examiners for this study.

### 2.2 Data Recording and Analysis

First, a single slice TIFF image was generated by the examiners from each original DICOM CT image data set at a level that included the lumen and both tympanic bullae at the mid‐point of the rostro‐caudal dimension of the bulla lumen. The TIFF format was used as it is a lossless format. If an image could not be generated with a visible bulla or lumen bilaterally due to asymmetric head positioning, the case was excluded from the dataset. In a second step at a later date, the same examiners (who made the initial data selection) determined the disease status of the subjects, and this was reviewed by a board‐certified radiologist. In case of disagreement, a consensus was reached and used as a diagnosis. The examination criteria for the nondiseased subjects required that the tympanic bulla be air‐filled, have a thin bony wall, and have a thin, straight tympanic membrane. For the diseased subjects, examiners checked if the tympanic bulla was filled with fluid, soft tissue, or mineralized material, if the tympanic membrane was not visible or bent (inwards with external ear disease, outward with middle ear disease), and if the tympanic bulla wall was either thickened or osteolysed. The diseased classification included either bilateral or unilateral evidence of middle ear disease.

The data scientists selected images at random and allocated them into training (74%), validation (13%), and test (13%) groups, with the number of normal and diseased cases in each group shown in Table 1.

**TABLE 1:** The number of normal and diseased CT images in the training, validation, and test sets, obtained by randomly splitting the canine dataset, while also ensuring a minimum number of examples for each class in the test set.

	Train	Valid	Test	Total
Normal	311	55	36	402
Disease	82	15	36	133
Total	393	70	72	535

#### 2.2.1 Data Preprocessing

The data scientists preprocessed the data using several steps, as follows. First, as the images contain patient metadata (e.g., date of acquisition and anonymized patient ID) embedded in both the DICOM and TIFF images, before any analysis was performed, this information was cropped out, and only the parts of the image relevant to training the model were included. Cropping was performed manually, prior to any rescaling or image manipulation, to include only the head image. Cropping at this point was variable in size, as images were later cropped and resized to a standard input size for the model. Next, the TIFF image data were normalized by subtracting the pixel mean and dividing by the standard deviation. Normalization standardizes the data and ensures that each image has a similar range of pixel values.

#### 2.2.2 Model Structure

The data scientists used a ResNet‐18 [10] CNN, with 5 blocks of 18 layers, with approximately 11 million trainable parameters (i.e., weights) in total. Deep learning approaches such as the one employed require large amounts of labeled training data. Given that our training dataset was relatively modest in size, we made use of transfer learning to improve the model's performance. The central idea is that instead of training the model from scratch on only the provided CT images, we make use of an initial model that has been pretrained on a large, diverse image classification task, for example, ImageNet [13]. The assumption of transfer learning is that the representation learned on this original larger dataset can be adapted to a downstream task of interest with minimal additional supervision (i.e., fine‐tuning). We tested two different transfer learning approaches: (1) retaining the original model weights from the initial ImageNet pretraining task but only training the final classifier, and (2) starting with the same pretrained model weights but fine‐tuning all the model weights on our training set. Method 1 is referred to as “FE” and Method 2 is referred to as “FT” in the result tables of this paper. Method 1 only requires updating a relatively small percentage of model weights, while Method 2 requires updating all the weights in the model.

#### 2.2.3 Data Augmentation

Data augmentation refers to a set of techniques that can generate additional data to increase the size and quality of the training set used for training deep learning models. It can be categorized into static or dynamic augmentation, depending on whether the dataset is expanded in advance. Static augmentation transforms the existing dataset once offline before training and stores it on disk, while dynamic augmentation uses a generator that transforms the data on‐the‐fly during training. In this study, both dynamic and static data augmentation strategies were investigated, with both strategies using the same methods and parameters as shown in Table 2.

**TABLE 2:** Parameters of the data augmentation methods used during training.

Method	Parameters
Grayscale processing	Grayscale (num output channels = 3)
Geometric transformation	HorizontalFlip (0.5), VerticalFlip (0.3), Rotation (45)
Color space transformation	Brightness (0.2), Contrast (0.2), Saturation (0.5)

Resampling and cost‐sensitive learning [14] were also considered to alleviate the problem of the imbalanced data, as the number of negative cases outweighs the number of positive (i.e., diseased) cases in the dataset. Resampling was performed when creating training batches by selecting more examples from the minority class to ensure that the number of examples provided to the classifier for the diseased and nondiseased classes is balanced. Cost‐sensitive learning was achieved by applying class‐specific weights to the training cost function such that incorrect predictions for the under‐represented diseased class were weighted higher by the loss function during training. The values of the class weight setting for each model are shown in Table 3.

**TABLE 3:** The summary of experiment models in this study.

Model	TL	DA	CW	OS
Baseline	No	No	No	No
FT_01	Fine‐tuning	No	[2.5,1]	Yes
FT_02	Fine‐tuning	Static	[2.5,1]	Yes
FT_03	Fine‐tuning	Dynamic	No	Yes
FT_04	Fine‐tuning	Dynamic	[2.5,1]	No
FT_05	Fine‐tuning	Dynamic	[2.5,1]	Yes
FE_01	Feature‐extractor	No	[1.8,1]	Yes
FE_02	Feature‐extractor	Dynamic	No	Yes
FE_03	Feature‐extractor	Dynamic	[1.8,1]	No
FE_04	Feature‐extractor	Dynamic	[1.8,1]	Yes

*Note*: For CW, the tuple given in the table is the label weights used by the loss function during training, whose first element is used for the diseased case and the second element is used for the normal case.

Abbreviations: TL, transfer learning; DA, data augmentation; CW, class‐weighting; OS, oversampling.

#### 2.2.4 Model Training

The Microsoft Windows‐based computer system used in this study had an Intel i5‐8365U 1.60 GHz CPU with 8 GB of RAM, while all model training was performed on a GPU cluster containing Nvidia RTX A6000 GPUs. All the experiments were implemented in the Python programming language using external libraries, including NumPy [15], PIL [16], and PyTorch [17]. The pretrained version of ResNet‐18 used in this study was obtained from PyTorch and was originally trained on the ImageNet dataset. In contrast, the baseline model used a ResNet‐18 without any pretraining, where all weights in the network were randomly initialized.

The input images were converted to grayscale, and then the smallest edge of each image was resized to 256 pixels, maintaining the original aspect ratio, and then a center crop of 224×224 pixels was extracted before being passed to the CNN. The model outputs are the binary classification score indicating the presence or absence of the disease. During training, models used mini‐batch updates with a fixed batch size of 16 and were trained using stochastic gradient descent (SGD) as the optimizer and with a binary cross‐entropy loss function. We used the same hyperparameters for all models, using values selected via initial experiments. The number of training epochs was set to 30, where one epoch corresponds to one pass over the entire training set. The initial learning rate was set to 0.0008, with momentum of 0.9, and was decreased by a factor of 0.95 after every five epochs. To prevent overfitting, models were selected based on the epoch with the greatest accuracy on the validation set, with a minimum training duration of five epochs. While it is possible to base the selection on other performance metrics, we selected accuracy as it is representative of real‐world evaluation.

#### 2.2.5 Evaluation of Model Performance

The data scientists evaluated model performance on our test set using several standard metrics for binary classification. Accuracy (Acc) measures the ratio of correct predictions over the total number of outcomes, Precision (P) measures how well the model can correctly identify the positive samples from the total predicted positive samples, Recall (R), also known as sensitivity (Sen), is equal to the true positive rate (TPR), which measures the proportion of positive samples that are correctly classified. Specificity (Spe) is equal to the true negative rate (TNR) and measures the proportion of negative samples that are correctly classified. The F1 score is the harmonic mean between the recall and precision values. The area under the Precision‐Recall curve is known as AP, and the area under the receiver operating characteristic (ROC) curve is known as AUC.

## 3 Results

The examiners classified 402 images as normal and 133 as diseased, including either bilateral or unilateral evidence of middle ear disease. The diagnostic imaging residents and the boarded radiologist obtained similar scores for all but a few borderline cases.

The data scientists compared the performance of 10 different CNN model variants as shown in Table 3. These included five fine‐tuning‐based transfer learning models, four feature extractor‐based transfer learning models, and a baseline model trained from scratch without any pretraining.

Based on the hardware described above, the training process for each model took approximately 40 min. Table 4 outlines the performance of all models defined in Table 3 on the test set evaluated using the metrics defined above.

**TABLE 4:** Summary of the performance on the test set of the developed models. Model FT_05 (in bold) achieves the greatest overall accuracy, with a balance between good sensitivity and high specificity.

Model	Accuracy	Precision	Recall/Sensitivity	Specificity	F1‐score	AP	AUC
Baseline	77.78%	0.955	0.583	0.972	0.724	0.86	0.87
FT_01	75.00%	0.909	0.556	0.944	0.690	0.88	0.86
FT_02	80.56%	0.923	0.667	0.944	0.774	0.86	0.86
FT_03	81.94%	0.960	0.667	0.972	0.787	0.89	0.89
FT_04	79.17%	0.800	0.778	0.806	0.789	0.90	0.89
**FT_05**	**84.72%**	**0.963**	**0.722**	**0.972**	**0.825**	**0.90**	**0.88**
FE_01	73.61%	0.870	0.556	0.917	0.678	0.86	0.81
FE_02	75.00%	1.000	0.500	1.000	0.667	0.85	0.79
FE_03	70.83%	0.941	0.444	0.972	0.604	0.80	0.76
FE_04	73.61%	1.000	0.472	1.000	0.642	0.85	0.81

*Note*: Here, FT and FE models are initialized using ImageNet weights, but only the final layer is trained for FE models, whereas all the layers are fine‐tuned for FT models. The Baseline model instead starts from randomly initialized weights.

The best automatic classifier FT_05, which was based on fine‐tuning using transfer learning, with dynamic data augmentation, oversampling, and class‐weighting, outperformed the baseline model on the test set, obtaining an accuracy of 84.7% (72.2% for disease cases and 97.2% for normal cases) compared with 77.8% for the baseline model. Furthermore, model FT_05 obtained an 8.9% improvement in accuracy, a 4.7% improvement in average precision, a 23.8% improvement in recall, and a 14.0% improvement in F1‐score compared with the baseline model. The baseline approach of training from scratch outperformed the feature extractor‐based transfer learning approach, while the end‐to‐end fine‐tuning approach outperformed both in terms of the overall model performance.

**FIGURE 3:**
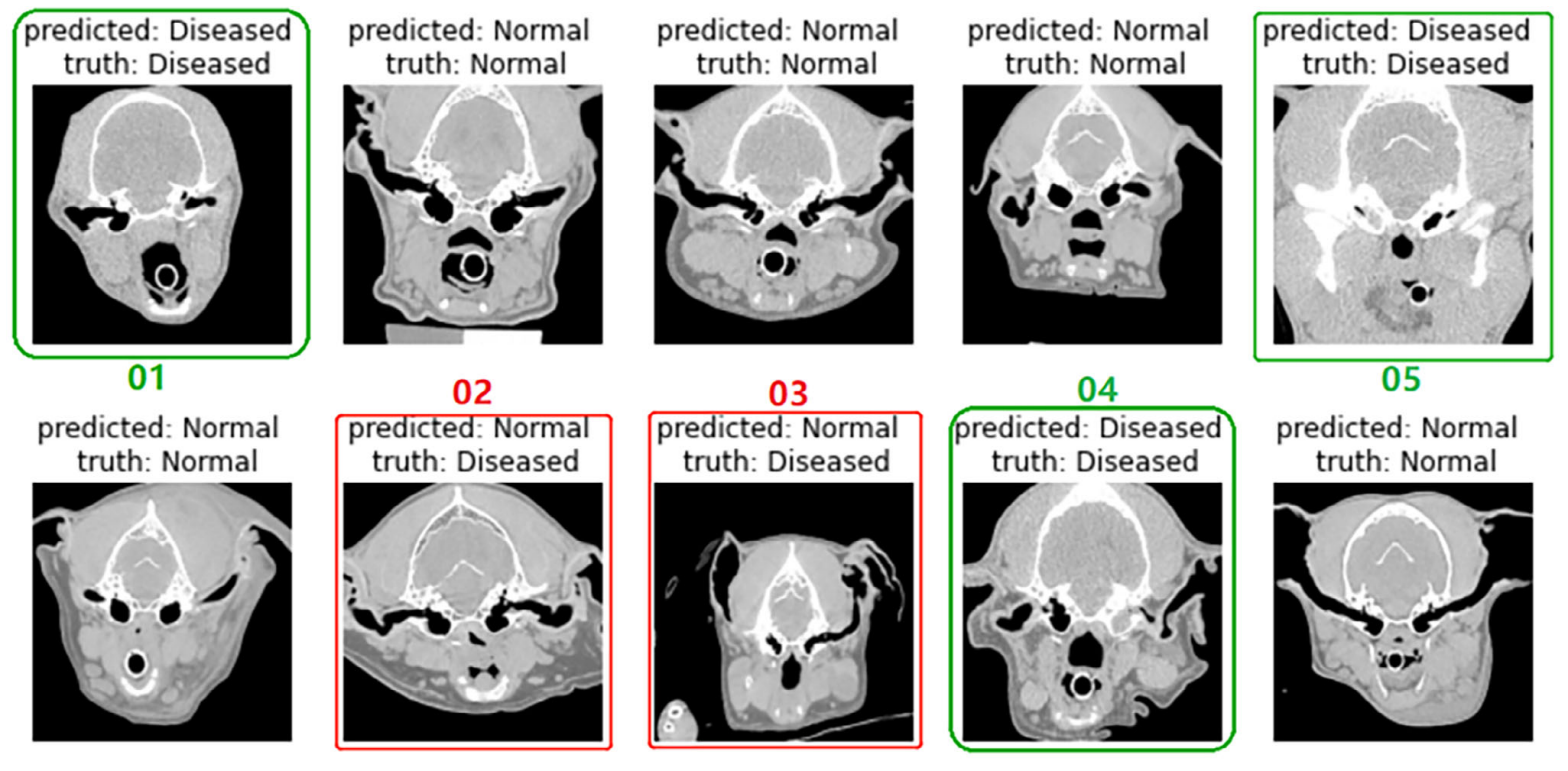
Examples of the prediction results of model FT_05 on a subset of images from the test set. Incorrect predictions are shown with red boxes, and correct predictions are highlighted using green boxes.

As an example, we visualized the prediction results of model FT_05 on 10 randomly selected, anonymously numbered samples from the test set in Figure 3. The model obtained correct results in 8 out of 10 cases.

## 4 Discussion

In this study, we have confirmed hypothesis (1), that a CNN can be trained to classify middle ears as normal or affected by disease. Whilst the accuracy is short of that of a trained radiologist, the emphasis here is on training a model with only a small number of training instances. However, the model can be considered to have a reasonable degree of accuracy despite this limitation, and it could be considered for use in an aided‐diagnosis setting. Despite the small training set, we have shown that data augmentation and transfer learning can partially alleviate this problem, and that class imbalance can be accounted for by weighting and oversampling, confirming hypothesis (2).

Modern deep learning approaches require large quantities of supervised training data to generalize to unseen test examples. However, a common challenge in automated medical image analysis is that it can be difficult to acquire the necessary large number of training examples (i.e., number of patients) for each disease of interest. If there is insufficient data, training a deep learning model becomes difficult. To address this problem, we explored different techniques (i.e., transfer learning techniques and data augmentation) to attempt to improve the developed classifier, considering the limited training data available. According to the results of this study, model FT_05 (with data augmentation) is 13.0% more accurate in relative terms than model FT_01 (without data augmentation), supporting our second hypothesis.

Perhaps unsurprisingly, our results demonstrate that using models that are trained on natural images as feature extractors, without adapting them to the CT images (i.e., the FE models), performs worse than fine‐tuning end‐to‐end (i.e., the FT models or baseline). This is due to the difference in the appearance of the web‐sourced images in ImageNet compared with our CT scans.

Another issue that arises in the context of medical data is that data are often imbalanced [18], that is, there can be many more examples from one class (diseased/normal) compared with the other. Unless this is addressed, there is a real danger that trained models can be biased toward the most common classes. This imbalance leads to a high probability of misclassifying diseased cases as being normal, which could result in patients not receiving timely treatment [19]. Consistent with our hypothesis, the two strategies (oversampling and class weighting) are useful for mitigating this problem. It should be noted that increasing the class weighting (and thus the importance) of the rarer diseased classes can increase the rate of false positive predictions from the model. This should be taken into account, in general, when training diagnostic tools where the number of negative cases often outweighs the positive cases.

A limitation of our study is that it is based on veterinary radiologists’ opinions as a gold standard instead of using confirmatory tests, such as otoscopy, culture, or histology. Whilst this would be interesting to explore, this was beyond the scope of our study. Another limitation was inherent in the data collection and scoring by the veterinary radiologists. The radiologists reviewed the entire DICOM set to select the single TIFF image and, by doing so, gained more diagnostic insights than the model had access to when training. This was partially mitigated by classifying at a separate time point. The head position was not always perfectly symmetric, which made assessment of both middle ears more challenging for both the examiners and when training the model. This is, however, a realistic approach to CT diagnostics and tests the robustness of the diagnostics. Finally, all preprocessing and training were done on the TIFF images. We believe the downsampling from DICOM to TIFF is unlikely to have any great effect, especially as the images are always further downsampled as part of the training pipeline.

There are some further limitations that can be explored in future work. First, the training data are quite limited, but it could be possible to scale the dataset with additional data sources to better improve the classification model. Second, the developed classifier uses 2D CT scan images from only two machines as input. Compared with this, 3D (volumetric) CT scans, and from a greater variety of machines, would likely provide more information about the middle ear structure and are thus potentially a better‐quality data source. In addition, only traditional data augmentation methods were applied in this study. In the future, learning‐based augmentation methods could be explored during training to enhance the performance of the model further.

## 5 Conclusion

In summary, this study examined the impact of different deep learning techniques for classifying middle ear disease in dogs. It is evident that the models with data augmentation at training time perform better than those without in almost every aspect. This is expected as data augmentation applies to all classes of images, including diseased and normal; therefore, it does not merely improve the model's ability to identify one class. Additionally, a combination of oversampling and class‐weighting outperforms either single method in isolation.

With this relatively small study, we contribute further evidence that, even with small‐scale datasets, classifiers can be constructed to aid in diagnosis. With a concerted effort to curate and annotate larger datasets in the veterinary radiology field, further improvements could be made, and the advances seen in AI‐assisted medical diagnosis could be translated into the veterinary space.

## Author Contributions

### Category 1

- Conception and design: Mac Aodha, Schwarz, Mellanby, Banks.
- Data acquisition: Daniel, Israeliantz, Orekhova, Schwarz.
- Data analysis and interpretation: Zhao, Daniel, Israeliantz, Orekhova, Schwarz.

### Category 2

- Manuscript drafting: Zhao, Banks.
- Review article for intellectual content: Zhao, Mac Aodha, Daniel, Israeliantz, Orekhova, Schwarz, Mellanby, Banks.

### Category 3

- Final approval of the completed article: Zhao, Mac Aodha, Daniel, Israeliantz, Orekhova, Schwarz, Mellanby, Banks.

## Conflicts of Interest

Authors followed the Strobe‐VET network guideline disclosure and the CLAIM guidelines. RM is currently employed by IDEXX Laboratories, Inc., in which he holds stock options. The remaining authors declare no conflicts of interest.

## Reporting Guideline Disclosure

The CLAIM reporting guideline checklist was adhered to in the preparation of this paper.

## Ethical Animal Research

The experimental data of this study is provided by the Royal (Dick) School of Veterinary Studies at The University of Edinburgh. The study protocol and data usage have been approved by the institutional veterinary ethical review committee (reference VERC 45.22).

## Previous Presentation or Publication Disclosure

This work has not been previously published, but initial results were orally presented at the BBSRC AI in Bioscience workshop, May 2021.

## Data Availability

Image data used in this paper is held by the Royal (Dick) School of Veterinary Studies at The University of Edinburgh. The dataset includes sensitive patient data. Access may be negotiated by contacting the corresponding author.
